# Multiple Sclerosis—Related Dietary and Nutritional Issues: An Updated Scoping Review with a Focus on Pediatrics

**DOI:** 10.3390/children10061022

**Published:** 2023-06-07

**Authors:** Claudia Mandato, Angelo Colucci, Roberta Lanzillo, Annamaria Staiano, Elena Scarpato, Luigi Schiavo, Francesca Felicia Operto, Maria Rosaria Serra, Cristina Di Monaco, Julia Sara Napoli, Grazia Massa, Pietro Vajro

**Affiliations:** 1 Department of Medicine, Surgery and Dentistry “Scuola Medica Salernitana”, Pediatrics Section, University of Salerno, 84081 Baronissi, Salerno, Italypietro.vajro@gmail.com (P.V.); 2 Multiple Sclerosis Clinical Care and Research Centre, Department of Neuroscience, Reproductive Science and Odontostomatology, University of Naples Federico II, 80138 Naples, Naples, Italy; 3 Department of Translational Medical Science, Section of Pediatrics, University of Naples Federico II, 80138 Naples, Naples, Italy; 4 Department of Medicine, Surgery and Dentistry “Scuola Medica Salernitana”, Nutrition Section, University of Salerno, 84081 Baronissi, Salerno, Italy; 5 Department of Medicine, Surgery and Dentistry “Scuola Medica Salernitana”, Pediatric Psychiatry Section, University of Salerno, 84081 Baronissi, Salerno, Italy

**Keywords:** multiple sclerosis, pediatric-onset multiple sclerosis, nutrition, diet, gut microbiota, gut-brain axis, blood-brain barrier, vitamin D, polyunsaturated fatty acids

## Abstract

Purpose. Lifestyle/dietetic habits play an important role in the development and progression of multiple sclerosis (MS) disease. Here, we examine the basic pathomechanisms underlying intestinal and brain barrier modifications in MS and consider diets and dietary supplementations proposed over time to complement pharmacological therapies for improving disease outcome both in adults and in children. Methods. Scoping literature search about evidence-based findings in MS-related gut-brain axis (GBA) pathophysiology and nutritional issues at all ages. Findings. Data show that (1) no universal best diet exists, (2) healthy/balanced diets are, however, necessary to safeguard the adequate intake of all essential nutrients, (3) diets with high intakes of fruits, vegetables, whole grains, and lean proteins that limit processed foods, sugar, and saturated fat appear beneficial for their antioxidant and anti-inflammatory properties and their ability to shape a gut microbiota that respects the gut and brain barriers, (4) obesity may trigger MS onset and/or its less favorable course, especially in pediatric-onset MS. Vitamin D and polyunsaturated fatty acids are the most studied supplements for reducing MS-associated inflammation. Conclusions. Pending results from other and/or newer approaches targeting the GBA (e.g., pre- and probiotics, engineered probiotics, fecal-microbiota transplantation), accurate counseling in choosing adequate diet and maintaining physical activity remains recommended for MS prevention and management both in adults and children.

## 1. Introduction

Multiple sclerosis (MS) is a disabling immune-mediated demyelinating neurodegenerative disease with an estimated prevalence of 1 in 1000 in populations of European descent. It primarily affects females (F:M = 2–3:1) mainly between the ages of 15 and 55 years. Pediatric-onset MS (POMS) is less common, and specific data for this age group are limited due to sample size and varying levels of functionality and cognitive maturation [1,2].

**Figure 1:**
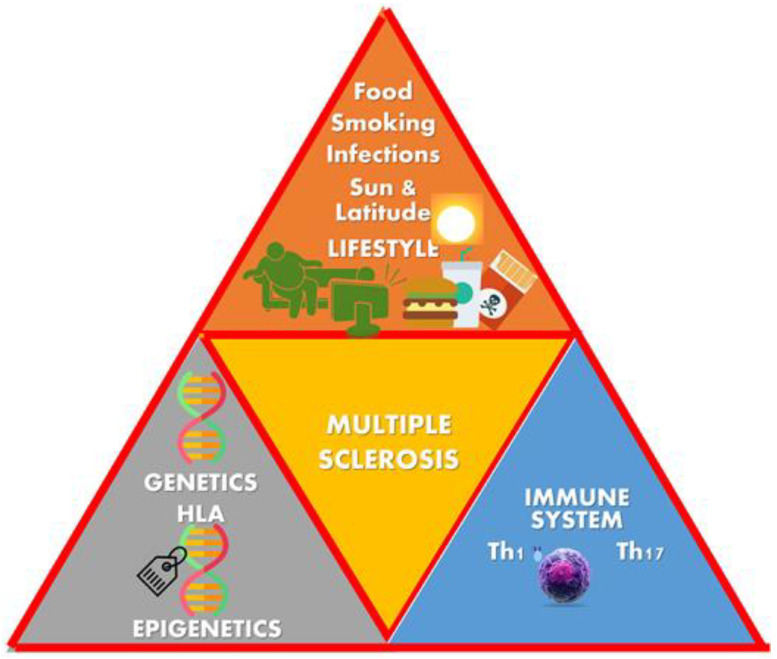
Multifactor etiology of multiple sclerosis (MS) [4].

The disease results from a complex interplay of immunological, genetic, and environmental factors [3,4] (Figure 1).

Studies in both murine models and patients suggest that an autoimmune CD4+ T cell-mediated inflammatory reaction initiates the demyelination process. These activated T cells, triggered by an unknown antigen, can cross the blood-brain barrier (BBB) and initiate an immune attack on the central nervous system (CNS). Inflammatory mediators released by T cells damage myelin, axons, and oligodendrocytes by recruiting and stimulating other immune cells such as B cells. This cascade leads to the formation of scar tissue (sclerosis) when myelin and nerve fibers are destroyed [5]. Genetic factors, particularly within the major histocompatibility complex (e.g., HLA DRB1*1501), contribute to about 50% of the risk of developing MS, although familial MS accounts for less than 15% of cases [5,6]. Environmental factors such as geographic location, infections (e.g., Epstein–Barr virus), smoking, toxins, nutritional deficiencies (especially vitamin D), sedentary lifestyle, unbalanced diets, changes in the gut microbiome, and pediatric-onset obesity have been implicated as risk factors for MS [7]. Recent evidence suggests a significant increase in the incidence of MS worldwide, particularly in Western countries, highlighting the importance of environmental factors in its pathogenesis [8]. Nutritional and dietary factors have gained increasing attention as potential modifiers of MS pathophysiology, aiming to complement current disease-modifying treatments (DMT) targeting inflammation and immune cells [9,10,11,12].The gut-brain axis (GBA), which is closely linked to nutrition and obesity, is also emerging as a critical player in MS [13]. This review aims to examine in-depth the latest growing literature on nutritional and dietary aspects contributing mechanistically to MS with a focus on their potential role as disease modifiers. Additionally, relevant emerging data pertaining to pediatric age will be reviewed to assess whether, as in adult MS, POMS disease management might benefit from add-on nutritional interventions.

## 2. Methods

This narrative review considered English language scientific literature articles published in the last 5 years, retrieved from the PubMed/Scopus and Google Scholar databases including observational and interventional studies, as well as both systematic and non-systematic reviews dealing with the subjects of nutrients/nutrition/diet/gut-brain-axis/blood-brain barrier/multiple sclerosis/pediatric-onset multiple sclerosis. Documents released as “grey literature” by the most relevant international health agencies and scientific associations made available on multiple electronic scoping searches as of April 2023 and relevant references quoted in the retrieved articles were also considered.

## 3. The Gut-Brain Axis in MS

The MS GBA contemplates a dysbiosis-induced pro-inflammatory gut environment responsible for a leaky gut [14] allowing the emergence of activated myelin-specific bystander T cells which regulate the cytokine milieu in the central nervous system (CNS) and the function of neurons and glial cells [13]. Clinical [relapsing-remitting (RR) course with subsequent progressive disability] and pathological similarities between MS and the murine model of experimental autoimmune encephalomyelitis (EAE) allow the latter to be considered as a suitable model for the study of MS. Through this model, studies have confirmed that high-fat diets, especially if high in saturated fats, are relevant MS triggers [15,16]. Obesity itself, due to the adipose tissue‘s inflammatory properties mediated by adipokines production (e.g., adiponectin), has been suggested to contribute to the pro-inflammatory status of MS, which also has impacts on the quiescence of CNS-resident microglia [17,18].

### 3.1. The Intestinal Barrier

The intestinal barrier is a complex functional unit composed of mucosal and luminal elements (i.e., epithelial cells layer; mucosal barrier; innate and acquired immune components); neuroenteric, vascular, and endocrine systems; digestive enzymes; and gut microbiota (GM). This barrier plays a key role in protecting against enteric organisms, their toxins, and bio-products associated with the health and disease susceptibility of organs/systems [19,20]. The recently discovered gut-vascular barrier controls the translocation of gut bacteria and antigens into the bloodstream [21,22].

The gut-(liver)-brain axis connects the GM, neuroendocrine and neuroimmune systems, autonomic nervous system, and enteric nervous system with the CNS [13,23]. The GM consists of trillions of commensal microorganisms that maintain the integrity of the mucosal barrier and contribute to normal host physiology [19]. Factors such as an unbalanced diet, infections, antibiotics, stress, and environmental factors can lead to dysbiosis and increased intestinal permeability [24,25,26]. Dysbiosis is associated with various diseases, including gastrointestinal and systemic inflammatory diseases [27].

GM dysbiosis can also impact the onset and progression of neurological disorders such as MS by affecting metabolic pathways and interacting with host immunity [28,29,30,31,32].

Identifying a characteristic/diagnostic composition of microbial communities associated with the gut microbiota of MS patients is challenging due to variations between studies and individual profiles [31,32]. However, reduced microbial diversity is commonly observed, which is characterized by an increased Firmicutes/Bacteroides ratio and prevalence of species producing endogenous ethanol, lipopolysaccharide, and reactive oxygen species [33,34].

These biochemical factors, along with pro-inflammatory T-helper types and cytokine patterns, contribute to intestinal inflammation and impairment of the barrier function (leaky gut) [29,35].

In an MS mouse model, Streptococcus thermophilus ST285 has been observed to switch cytokine responses to myelin peptides from pro-inflammatory to anti-inflammatory patterns [36]. The significance of increased abundance of Akkermansia species in MS patients and the EAE mouse model is still debated, with suggestions of a compensatory effect rather than a direct association with EAE progression and MS pathogenesis [37,38].

Considering differences in patient selection methods, environmental factors, and dietary habits, certain species (e.g., Faecalibacterium, Eubacterium rectale, Corynebacterium, Fusobacteria, Bacteroides stercoris, and Bacteroides coprocola) [29,39] are generally reduced in MS patients compared to healthy controls in some studies. These species are responsible for decreased production of metabolites such as bacterial lipid 654, a Toll-like receptor 2 ligand derived from gastrointestinal and oral bacteria [40,41,42], and short-chain fatty acids (SCFAs) such as butyrate and propionic acid [14,43,44].

SCFAs, produced by the colonic fermentation of dietary fibers and resistant starch, are speculated to play a key role in neuro-immunoendocrine regulation [45]. A secondary increase in species related to oxidative levels is observed in progressive MS or more severe disease forms [46,47,48], and it is sufficient to induce EAE sensitivity [49,50]. Saturated fatty acids (FAs) have a close relationship with autoimmunity phenomena in MS, as their concentration and composition regulate immune cell polarization, differentiation, and function, with a protective role in blood-brain barrier function. They are critical players in CNS chronic inflammation, progressive degeneration, and remyelination [34,51].

Additionally, a negative association with polysaccharide-digesting bacteria such as B. thetaiotaomicron is often observed [52].

### 3.2. The Blood-Brain Barrier

**Figure 2:**
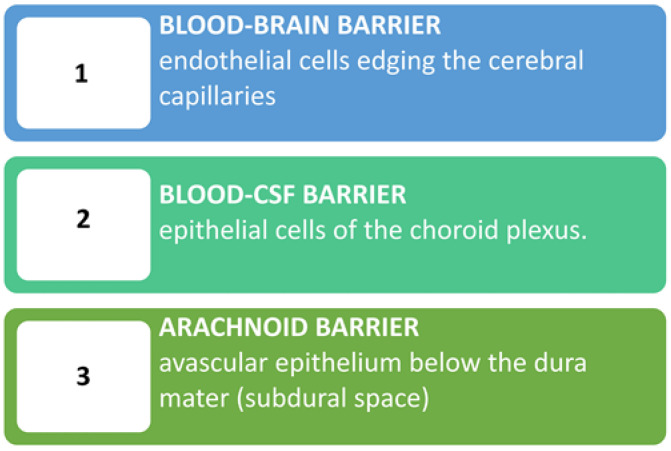
The blood-brain barrier consists of biological barriers formed by different cells at three key interfaces. The 1st layer is made up of microvascular endothelial cells that line the cerebral capillaries and permeate the brain and spinal cord. The 2nd barrier is made up of the epithelial cells of the choroid plexus. This layer, being more permeable to proteins thanks to the presence of a fenestrated endothelium below a cuboidal epithelium, may regulate brain permeability under conditions of gut inflammation. The 3rd barrier is situated below the dura mater and contributes little to blood-brain exchange due to its avascular nature and relatively small surface area compared to other barriers. CSF: Cerebrospinal fluid.

The BBB plays a crucial role in maintaining the homeostasis of the brain microenvironment, and its dysfunction is implicated in the pathogenesis of various neurological diseases, including MS. The BBB consists of multiple biological barriers including the proper blood-brain barrier, the blood-cerebrospinal fluid barrier, and the arachnoid barrier [22,53] (Figure 2).

The integrity of the BBB can be influenced by the GM, as bacterial antigens such as lipopolysaccharide (LPS) and SCFAs can travel from the leaky gut to the brain endothelial cells’ Toll-like receptors 2 through the bloodstream. In the EAE murine model, high-fat-diet-induced obesity resulted in severe disease accompanied by gut dysbiosis, increased gut permeability, and systemic inflammation, suggesting a role for gut barrier modulation in obesity-induced MS severity [39]. In a similar EAE model, obesity induced by a high-fat diet also led to BBB disruption, which allows the infiltration of monocytes/macrophages and activation of resident microglia, ultimately exacerbating CNS inflammation in EAE [54], likely mediated by IL-6 and CCL-2 [55].

The activation of pro-inflammatory pathways disrupts the delicate balance between protective and harmful reactions. It can induce the release of Vascular Endothelial Growth Factor B/VEGF-B and Tumor Growth Factor/TGF-alpha from microglia, which activates astrocytes and exerts detrimental effects on neurons. The Th17 cytokines IL-17A and IL-17F appear to be pivotal in triggering BBB disturbance [56,57]. As mentioned earlier, SCFAs not only have effects on the colon and peripheral tissues but also play a crucial role in the communication between the GM, gut, and brain. They can cross the BBB through endothelial cell monocarboxylate transporters and a) upregulate the expression of tight junction proteins essential for BBB integrity, b) affect neuroinflammation by influencing glial cell morphology and function, and modulating levels of neurotrophic factors [45]. Moreover, due to its bidirectional nature, GBA neuroinflammation could result in further intestinal inflammation as the disease progresses by affecting efferent cholinergic transmission [13,58,59].

## 4. Diets and Dietary Supplementations

### 4.1. Dietary Influence on MS

Studies in adults with MS suggest that diet-related inflammation increases the odds of developing the disease [60,61]. The prevalence of Westernized diets, characterized by ultra-processed foods that are high in salt, saturated and trans fatty acids [62] and low in fibers and flavonoids, may contribute to the upregulation of pro-inflammatory compounds, gut dysbiosis, neuroinflammation, and neurodegeneration [63,64,65]. The high salt content in processed foods has been associated with disease exacerbation and the development of new lesions, although the evidence is debated [66,67,68]. Saturated fats activate pro-inflammatory Toll-like receptors (TLRs) and increase NF-κB, which affects the innate immune system [7]. They also play a role in GM-mediated inflammation in MS development and relapse risk, as seen in the EAE model [39,54,55] or in MS patients. [63,65,69].

Conversely, higher fish consumption, particularly oily fish rich in vitamin D and omega-3 fatty acids, is associated with a lower risk of CNS demyelination [70]. Flavonoids, polyphenolic compounds that are abundant in fruits and vegetables, have shown a protective role in MS development in experimental models [71,72]. Non-fermentable cellulose fiber may prevent changes in GM composition and T cell responses associated with CNS autoimmunity in MS [50]. The proper functioning of all players in the gut-brain axis is crucial for managing the impact of MS [Table 1].

Several diets have been proposed for MS based on assumptions such as existing food allergies, gluten sensitivity, hypovitaminosis, or the concept of a healthy diet (e.g., Mediterranean diets, Paleolithic diet) [73]. Although a diet rich in fruits and vegetables seems logically protective against relapses and disease progression [74], larger and better-conducted studies are needed to confirm this correlation due to conflicting evidence [75].

**Table 1:** Synopsis of the main specific diets proposed over time in patients with multiple sclerosis.

Diet Name	Main Characteristics
Allergen free/milk free	Hypoallergenic diet based on the unproven hypothesis of the association between MS and external allergens [76]. The milk protein butyrophilin has been implicated through antigenic mimicry with myelin oligodendrocyte glycoprotein in EAE [77] as well as in MS patients [78]. Some studies with questionnaires suggest an inverse relationship between total dairy intake and MS disability severity [79,80] with an inverse relationship between whole grain intake and MS-related disability [80].
Gluten free	Among studies, only one clinical trial gave meaningful results, but there are methodological limitations [81,82,83]. All in all, the current level of evidence is inadequate to state whether gluten plays a role in MS [82].
Mega Ascorbic	High in vitamin C diet. No well-defined link between MS and vitamin C [84].
Multi Vitaminic	Multi vitaminic supplementation (e.g., A and D): quite convincing data show that higher vitamin intake/serum levels correlate with lower risk of MS development but not convincing on the contrary [85]. Possible detrimental effects of overdosing require vitamin-level monitoring [86,87].
Hebener’s	Self-reported disease stability/amelioration in one study with fish oil and antioxidant drugs supplementation + Ω-6 restriction [88].
Kousmine	High in polyunsaturated fats/low in animal fats diet to counteract a possibly increased membrane permeability [89,90,91].
Swank (low saturated fats)	Low-saturated fats (<20 g fat/day or <20% total calories): reported lower death rates and better outcome in the more adherent patients and those with lower disability at entry [92,93].
Mediterranean diets (MD)	Common features include emphasis on vegetables, fruits, beans, nuts, seeds, breads, unrefined grains, and olive oil; inclusion of fish and wine; minimal intake of full-fat dairy products and possibly lean meats [94,95]. Conflicting results on whether lean and unprocessed red meat is detrimental [96,97,98]. It is considered beneficial for its antioxidant properties. Negatively associated with neurological and fatigue symptoms. Adherence should be monitored through validated tests [e.g., Predimed for adults [94] and KidMed for children [99]
Mediterranean/DASH	It derives from the Mediterranean Dietary Approaches to Stop Hypertension (DASH) [100,101].
MIND	The Mediterranean/Intervention for Neurodegenerative Delay (MIND) is a combination of MD and DASH [100,101,102].
Paleolithic1	Consists of high-quality foods full of nutrients and fiber and with less artificial sugar and salt compared to present-day diets [103]. Nutrients included in this diet are essential to myelin growth and repair. Typically, it does not permit consumption of dairy or grain products.
Modified Paleolithic (MD-PI intervention)	This diet is rich in α-lipoic acid and polyphenols. It has commonalities with MD including avoidance of high-fat meats/ultra-processed foods with added sugar, sodium, and hydrogenated fats [104,105,106].
Wahls™ Paleo diet	Differences from a traditional Paleo diet: exclusion of eggs; limited animal and fish protein. It allows legumes (e.g., soy milk), two servings of gluten-free grains (e.g., rice) per week; it specifies nine cups of fruits and vegetables (F/V)/day with 1/3 each from dark-green leafy vegetables, sulfur-rich vegetables, and deeply colored F/V; seaweed, algae and nutritional yeast are encouraged [107,108].
Wahls/Elim Paleo	This is a paleo version modified by adding a restriction of lectins to reduce intestinal permeability and CNS inflammation [107,108].
Overcoming MS (OMS)	Minimized saturated fats and plant-based, whole-food diet plus seafood [79,109].
Ketogenic diet (KD)	Eliminating all/almost all carbohydrates and increasing the intake of proteins. KD combined with a modified MD have been suggested to improve neuroinflammation in MS [110,111].
Energy restriction (ER)	Chronic ER/Intermittent energy restriction (IER) determines a switch from glucose to fatty acids and ketones as the major fuel source for cells [112,113,114,115]. Mice fed a “fasting mimicking” diet (very low-calorie diet lasting for 3 days every 7 days) exhibited delayed onset, reduced incidence, and decreased severity of EAE. Histological findings show reduced immune cell infiltration and demyelination in the spinal cord [116].
McDougall Diet	A low-fat (10–15% of calories from fat), starch-based, vegan diet with no oils permitted. For 7 days, produced significant favorable changes in commonly tested biomarkers used to predict future risks for cardiovascular disease and metabolic diseases [75,117]. It appeared safe and effective in preventing clinical attacks/new MRI lesions. Drawback = long-term adherence [118].

Abbreviations: DASH, Dietary Approaches to Stop Hypertension; ER, Energy restriction; F/V, fruit and vegetables; KD, Ketogenic diet; MD, Mediterranean diet; MIND, Mediterranean/Intervention for Neurodegenerative Delay; MS, multiple sclerosis; OMS Overcoming MS; PI, Paleolithic intervention.

A recent meta-analysis [119,120] examined 11 studies (involving 608 participants) that investigated whole dietary approaches without concomitant interventions with appropriate control groups and outcome measurements. The modified Paleolithic and modified Mediterranean diets improved fatigue and quality of life compared to the control diets. Low-fat diets improved fatigue but not quality of life. Fasting, calorie-restricted, and anti-inflammatory diets did not significantly affect fatigue or quality of life, and ketogenic diets fell somewhere in between. GM appeared to be a pivotal element influenced not only by the type of diet but also by therapies. In fact, vitamin D supplementation, commonly prescribed for MS [121], may also influence gut bacterial populations by increasing the frequency of certain genera [122]. In vitro studies have shown that some MS drugs (e.g., fingolimod and teriflunomide) also have anti-inflammatory and antioxidant effects and can shape GM and inhibit the growth of neurotoxin-secreting gut bacteria. [123] Regular exercise has been shown to have positive effects on sleep, depression, paresthesia, fatigue, and cognitive performance [124,125]. However, the impact of physical activity on GM composition and microbial metabolites in the gastrointestinal tract in MS patients remains to be studied [126].

Other factors, such as socioeconomic status, quality of life, and personal motivation, may contribute to the uncertainty surrounding diet-related results. Adherence to an MS-specific diet is associated with higher socioeconomic status, better quality of life, and higher nutritional quality [127]. Enhancing personal motivation and ensuring positive support from study staff and family members are opportunities for future dietary intervention studies in MS, as they can improve adherence and reduce attrition [118,128]. Additionally, strategies should be developed to tailor study diets to the preferences of both individuals with MS and their household members to reduce feelings of burden and improve diet observance [118,128].

Progressive MS disabilities can impact grocery shopping, cooking, and eating, leading to weight loss, isolation, and dysphagia in advanced stages [129,130]. Proper nutritional support and guidance are necessary to ensure a correct diet at any age, starting from the early stages of the disease [120,131,132,133]. On the other hand, an unbalanced diet coupled with reduced physical activity can result in overweight/obesity, which triggers EAE and MS onset and progression [17,54,55,134,135,136,137,138]. Overweight and obesity are also associated with cardiovascular disease, which contributes to more rapid disability progression in MS [139,140,141].

Considering the role of GM in MS onset and progression, probiotics are of great interest [142]. Probiotic supplementation can modify GM composition and intestinal barrier function, potentially modulating GBA pathways, immune cells, and inflammatory cytokines. Studies in MS patients and animal models have shown promising but inconclusive results, including slower disability progression, reduced depressive symptoms, and improvements in general health [143,144]. Further research is needed to explore different strains and their effects on GM composition, as they may depend on ongoing diets and therapies [145,146,147,148].

### 4.2. Dietary Supplementations

Apart from probiotics, dietary supplementation of several compounds proposed to increase anti-inflammatory and antioxidant activities have been reviewed by a recent Cochrane metanalysis including 30 randomized controlled trials (RCT) or controlled clinical trials (CCT) among participants with MS on MS-related outcomes, i.e., relapses, disability progression, and magnetic resonance imaging (MRI) measures [149]. After reviewing dietary programs including supplementation to increase PUFAs [comparing PUFAs vs. MUFA or PUFA Omega 6 vs. Omega 3] and other dietary supplements (e.g., antioxidants, acetyl L-carnitine, biotin, creatine, riboflavin), the metanalysis concluded that, at present, there is insufficient evidence to determine whether supplementation with antioxidants or other dietary interventions has any real impact, whether beneficial or harmful, on MS-related outcomes [149,150]. These data confirmed others’ conclusions that the body of present evidence is primarily focused around the isolation of individual nutrients, many of which demonstrate no clear effect on major outcomes of MS progression [151]. Of note, although some uncertainties can depend on the dosages used and/or the duration of the treatments (e.g., PUFA), supplementations with other compounds (e.g., vitamin D) may depend on patients’ pre-existing nutritional adequacy as opposed to a need for high-dose supplementation [151]. Recent data confirm that although there are no statistically significant correlations between clinical outcomes and vitamin D serum levels or supplementations, patients receiving vitamin D had fewer new T2-weighted lesions, especially when optimal or higher levels of vitamin D (>30 ng/mL) were maintained throughout the entire 4-month observation period [152]. All in all, stronger studies focused on food and nutritional supplementation are required to strengthen the evidence.

## 5. Diet and Nutrition Related Issues in Pediatric-Onset MS

MS starting in childhood is estimated to account for between 2–5% and 5–10% of the MS population worldwide, with an estimated global incidence ranging from 0.05 to 2.85 per 100,000 children a year and a prevalence of 0.7 to 26.9 per 100,000 children. The median age of children with POMS is 12 years (range: 1.6–17). It is not clear whether the recently reported increased incidence may be real or more likely dependent on better diagnostic methods [2,153,154,155,156]. As outlined before, at any rate, data in POMS remain scarce mainly due to small sample size [1,157]. Despite POMS’s active inflammatory course, with frequent relapses/partial recovery episodes [158,159,160], presently available DMT may attain a discrete inflammation control [161,162], with a beneficial reduction of annualized relapse rates which may interfere with age specific cognitive impairment [160]. This is important because, although children and adolescents have a lower risk of disability within the first 10 years of diagnosis than those with adult-onset MS, in fact, the disease may negatively affect their school and emotional spheres [163]. Moreover, young patients reach disability milestones earlier than adults, even though they tend to take a longer time to advance to the secondary progressive phase [34,164,165]. Consequently, healthcare resource utilization and costs are high in this population compared with healthy children and adolescents. Quite common cognitive impairment requiring specific management, decrease in QOL, and an increase in economic burden in POMS have been shown to have profound impacts not only on patients but also on their families [154,155]. Still, an early onset of MS seems to be somewhat protective regarding QOL [165], maybe due to better resilience resources in youth [154,155,166]. Efforts towards a better understanding of possible specific mechanisms that trigger POMS and improving inherent therapies and prevention appear, therefore, to be necessary in young and pre-pubertal children [162,167,168].

Childhood obesity has been identified as a potential risk factor for increased morbidity not only from its hepatic-cardiac-metabolic comorbidities [169,170] but also from MS and clinically isolated syndrome (CIS) in adolescents, particularly in girls [134]. The underlying mechanism may involve vitamin D deficiency, as obesity is associated with lower vitamin D levels [136,171]. The association between excess weight and MS risk highlights the importance of addressing the childhood obesity epidemic and its potential impact on pediatric MS [171]. Of note, sedentary indoor lifestyles and reduced sunlight exposure, exacerbated by the COVID-19 pandemic, contribute to decreased vitamin D synthesis and increased hypovitaminosis D in children [172].

Dietary factors have also been studied in relation to MS outcomes in children. A study analyzing dietary intake in children with early-onset pediatric MS found that unbalanced diets with increased fats, especially saturated fat content, were associated with a higher risk of unfavorable disease progression [173]. However, sugar intake did not appear to be associated with a higher relapse risk [173]. Additionally, a healthy diet in childhood, characterized by the consumption of fruit, yogurt and legumes, was associated with a lower probability of developing MS in adulthood [174]. These findings suggest that specific dietary strategies may aid children with POMS in slowing disease progression and improving their quality of life [163].

Minerals, such as iron, may also play a role in POMS progression [175]. Iron deposition in gray and white matter has been observed in MS patients, and iron chelation therapy has been proposed as a potential therapeutic approach [176]. Conversely, iron deficiency may contribute to disease progression, as iron is involved in myelin synthesis and normal cellular metabolic pathways [177,178]. Further studies are needed to clarify the role of iron in POMS.

The gut microbiota (GM) has also been implicated in POMS. Differences in microbial composition and metabolic pathways have been observed in children with POMS compared to healthy controls [179,180]. GM composition may predict the likelihood of recurrence in pediatric MS [181,182].

Due to its effects on levels of disease activity, depression and fatigue, and lower moderate to vigorous physical activity, prevention and/or treatment of obesity/being overweight has been deemed a critical aspect of POMS as well [183]. A case-control study comparing the BMI data of 60 French youths with POMS showed that overweight and obesity are more frequently observed at diagnosis, particularly in boys with POMS compared with non-neurologic controls and healthy French children. Moreover, higher BMI is related to initial inflammation in the CSF in prepubertal patients with POMS suggesting an interaction between excess body fat, sexual hormones, and POMS occurrence [135]. These data are particularly worrying if one considers the findings that a large proportion of adolescents with POMS also have a non-self-perceived elevated BMI [184]. To improve their disease progression, they should therefore receive more accurate counseling to improve their diet [184] and physical activity as well [185].

## 6. Discussion

The last few years have been characterized by a mounting number of articles providing experimental and clinical evidence of the role of some nutrients and diets in MS. Most studies have regarded adult MS, focusing prevalently on risk factors rather than on disease progression, and there is still limited available evidence for many foods and nutrients [186]. Although more research is needed to fully understand their relationship with MS, vitamin D and PUFA appear to be the most relevant single molecules researched. Studies suggest that people with lower vitamin D levels have a higher risk of developing MS. In addition, research has shown that vitamin D supplementation can help reduce the severity and frequency of MS symptoms. Similarly, a diet high in PUFAs can help reduce the risk of developing MS and improve symptoms in people who already have the disease. Other relevant protective dietary factors could start during the early years, such as a prolonged duration of breastfeeding [187,188,189]. Regarding diets, many suggestions have been reported over time. Granted that no universal best diet exists, as it can vary based on patients’ individual needs and preferences, and that healthy and balanced diets are necessary to ensure adequate intake of all essential nutrients, some diets that have shown some beneficial effects typically share the conditions of having high intakes of fruits, vegetables, whole grains, and lean proteins while limiting processed foods, sugar, and saturated fat. All in all, these components have antioxidant and anti-inflammatory properties and tend to shape a healthy GM capable of respecting the gut and brain barriers. The effects of a Mediterranean diet on MS disability appear appealing but remain still uncertain [119,190]. Several other nutrition-related strategies have been proposed as an additional measure to DMTs. Among these, probiotics in the future will need further, better-conducted studies to confirm whether they can represent a viable chance as a personalized microbiota-based adjuvant therapy. Nondigestible dietary fibers (prebiotics) offer another related possibility to reshape altered GM in MS, which appears to be a practicable avenue warranting to be further explored. In fact, they contribute to gut eubiosis allowing the formation of microbial metabolites such as SCFAs in the colonic lumen with key immunological regulatory mechanisms in MS [29,191].

So far, nutritional and dietary data available on POMS are limited. However, they suggest that, as in adults, POMS’ disease management might benefit from an add-on nutritional intervention. In particular, it appears that a diet rich in vegetable intake and low in saturated fat may reduce the risk of relapse [173]. Studies on other interventions for this group are necessary, because simple translation of evidence targeting diet or nutrition available in adults is not always feasible, as it may be the case of salt intake [192,193,194,195]. Besides dietary factors, physical activity (PA) and sleep are the other two important health behaviors in both adults and youths with MS, as they represent a modifiable factor. In a scoping review of the available literature [195], the authors concluded that the PA self-reported by children with POMS was (a) generally lower than that of healthy controls and those with other types of demyelinating diseases, and (b) may be associated with markers of disease burden and symptomatology. Intriguingly, children with POMS performed better than matched controls in managing sleep hygiene, and higher levels of physical activity were associated with fewer sleep/rest fatigue symptoms [195]. It is noteworthy that, quite similar conclusions were also reached by other independent authors [183].

Overall, our review must take account of its limitations due to the fact that only a few of the considered studies were controlled and the enrolled patients who volunteered for randomization were obviously not blind. Both in adults and in pediatric age, most data derive from enquiry-based dietary/nutritional studies which have, as it may be expected, several drawbacks. These include the fact that food frequency questionnaires may be subject to recall-bias-driven report errors, lack of relevant food items and nutrients, and focus on a few days’ diet instead of real long-term dietetic habits. Moreover, their retrospective nature only allows them to shed light on an association and not on a causation [80,112,190]. On top of these issues, confounding conclusions may derive from interferences deriving from the real level of adherence to the diets [127,128,129,130,196], level of sleep [124,125], and the quite under-rated effects of associated PA [124,125,126], vitamin D [122] and some DMT [123] on GM.

Finally, new, yet unexplored therapeutical paths are mandatory to further complement the presently available therapies. What is around the corner? Next generation (NG) engineered probiotics, obtained by modifying original probiotics through gene editing modalities, have hitherto been used in inflammatory bowel disease, and in a number of bacterial infections, tumors, and metabolic diseases, mainly in EAE murine models and/or in vitro [197]. Promising preliminary results showing they are effective, with fewer side effects than traditional treatments or wild-type strains [198,199], suggest that they will probably be proposed soon for CNS diseases as well, including MS. Of note, it has been underlined that the design of NG probiotics should specifically be directed towards increasing the production of metabolites (e.g., SCFAs) and neurotransmitters (e.g., serotonin, GABA) which are known to affect the neurobiology of CNS inflammatory diseases [200]. Last but not least, fecal microbiota transplantation (FMT) represents a further interesting approach to modulate GM [201,202]. FMT studies in animal models and in humans with MS are still scarce and preliminary. Some data available from a cohort of RRMS patients show that FMT is safe and was well-tolerated and may have also improved their gut dysbiosis and elevated small intestinal permeability [203]. Moreover, single case reports and a case series (totaling six patients) reviewed by Matheson et al., in addition to confirming the safety of the treatment, also showed specific clinical improvements in MS-related neurological symptoms [204].

## 7. Conclusions

In conclusion, several dietary/nutritional factors play an important role in MS development and progression. Several gut-oriented nutritional interventions aiming to improve the dysregulation of the so-called gut-brain axis through a proper diet appear to intervene beneficially mainly against the inflammatory pathomechanisms associated with MS. Nutritional issues are therefore worthy of further research with high-quality, long-term, randomized studies in the global treatment of patients with MS [205]. The efficacy of any dietary intervention in MS, however, remains very difficult to prove due to spontaneous remissions (and relapses) with temporary clinical improvement occurring by chance alone. Pending more solid evidence on specific diets, experts suggest that individuals with MS should be taught to follow a “healthy” regime [128,190] and possibly enter into nutrition education programs, which, however, are largely lacking for patients with MS and most other neurological diseases at present [206]. Because of the high prevalence of overweight/obesity, and the evidence that obesity can worsen MS prognosis, education on weight management is still an unmet need, and non-guided patients are frequently no more likely to adopt a specific diet than normal weight patients [138]. Pediatric interventions may be even more hampered by the lack of self-perceived BMI elevation in MS youths [184,185]. Because there is, at present, no robust evidence, future research is also needed to identify appropriate study designs and intervention strategies targeting PA participation, with measurements of their outcomes on the primary and secondary symptoms of POMS [195]. New solid longitudinal and experimental designs are necessary not only to better elucidate the role of diet and other modifiable lifestyle factors in this population, but also to explore other modalities of support. These should include a closer monitoring of nutritional status of patients with moderate-advanced MS in order to prevent their tendency to be overweight secondary to the decrease in basal energy expenditure and loss of muscle mass. Additionally, the intervention of other health/psychology/social professionals should contribute to overcoming barriers and improving quality of life by avoiding MS patients’ and their relatives’ struggles with loneliness and rejection [132,133].

## Data Availability

Not applicable.
